# Hydrodynamics Alter the Tolerance of Autotrophic Biofilm Communities Toward Herbicides

**DOI:** 10.3389/fmicb.2018.02884

**Published:** 2018-12-04

**Authors:** Bastian H. Polst, Christine Anlanger, Ute Risse-Buhl, Floriane Larras, Thomas Hein, Markus Weitere, Mechthild Schmitt-Jansen

**Affiliations:** ^1^Department of Bioanalytical Ecotoxicology, Helmholtz-Centre for Environmental Research – UFZ, Leipzig, Germany; ^2^Institute of Hydrobiology and Aquatic Ecosystem Management, University of Natural Resources and Life Sciences, Vienna, Austria; ^3^WasserCluster Lunz, Lunz, Austria; ^4^Department of River Ecology, Helmholtz-Centre for Environmental Research – UFZ, Magdeburg, Germany; ^5^Institute for Environmental Sciences, University of Koblenz-Landau, Landau, Germany

**Keywords:** autotrophic biofilm, multiple stressors, microbial communities, PICT, near-bed hydrodynamics, periphyton

## Abstract

Multiple stressors pose potential risk to aquatic ecosystems and are the main reasons for failing ecological quality standards. However, mechanisms how multiple stressors act on aquatic community structure and functioning are poorly understood. This is especially true for two important stressors types, hydrodynamic alterations and toxicants. Here we perform a mesocosm experiment in hydraulic flumes connected as a bypass to a natural stream to test the interactive effects of both factors on natural (inoculated from streams water) biofilms. Biofilms, i.e., the community of autotrophic and heterotrophic microorganisms and their extracellular polymeric substances (EPS) in association with substratum, are key players in stream functioning. We hypothesized (i) that the tolerance of biofilms toward toxicants (the herbicide Prometryn) decreases with increasing hydraulic stress. As EPS is known as an absorber of chemicals, we hypothesize (ii) that the EPS to cell ratio correlates with both hydraulic stress and herbicide tolerance. Tolerance values were derived from concentration-response assays. Both, the herbicide tolerance and the biovolume of the EPS significantly correlated with the turbulent kinetic energy (TKE), while the diversity of diatoms (the dominant group within the stream biofilms) increased with flow velocity. This indicates that the positive effect of TKE on community tolerance was mediated by turbulence-induced changes in the EPS biovolume. This conclusion was supported by a second experiment, showing decreasing effects of the herbicide to a diatom biofilm (*Nitzschia palea*) with increasing content of artificial EPS. We conclude that increasing hydrodynamic forces in streams result in an increasing tolerance of microbial communities toward chemical pollution by changes in EPS-mediated bioavailability of toxicants.

## Introduction

Most aquatic systems suffer from exposure to multiple stressors. This may be the cause why most water bodies at the European scale fail to reach the ecological quality goal such as the “good ecological status” according to the EU-Water Framework Directive (EEA, 2012). Approximately two third of European rivers are affected by two or more stressors at the same time with water quality pressures (59% of European rivers) and alterations of hydrology and hydrodynamics due to channelization or impoundments (41% of European rivers) being widely distributed (Schinegger et al., 2012). Co-occurring stressors may interact, leading to non-linear and complex responses which are difficult to characterize and predict, but may have important implications for the management of aquatic systems (Côté et al., 2016). Whereas the co-occurrence and potential interactions of stressors were analyzed on a large scale (Côté et al., 2016), there is still limited knowledge on the mechanisms of stressor interactions on local communities. However, an improved understanding of stressor interactions is essential to manage and restore water bodies under multiple stresses. Chemical stressors are underrepresented in current surveys on multiple stressors (Nõges et al., 2016; Schäfer et al., 2016), but have been identified to pose a potential risk to more than 50% of rivers in Germany (Schäfer et al., 2016). Consequently, the co-occurrence and interactions of hydrodynamics and chemical stress in streams and rivers is very likely.

Biofilms play an important role in mediating essential functions and biogeochemical processes within aquatic ecosystem (Battin et al., 2003b, 2016). At the same time, they are especially vulnerable to chemical exposure and physical stress induced by hydraulic disturbance (Sabater et al., 2007; Larned, 2010; Romero et al., 2018). Often also referred to as periphyton, aquatic biofilms cover a wide range of different taxonomical and functional groups including fungi, bacteria, archaea, algae, protozoa, and viruses colonizing surfaces at and within the stream channel bed. Embedded in a matrix of extracellular polymeric substances (EPS), biofilm organisms shape their own microenvironment (Flemming and Wingender, 2010). They are highly dynamic and can rapidly adapt to changing conditions regarding pollution and flow velocity (Villeneuve et al., 2011a) making them suitable indicators for environmental change (Sabater et al., 2007).

In streams and rivers, hydrodynamics affect structural and functional parameters of biofilms. For instance, primary production and algal biodiversity as well as biofilm internal mass transport were reported to decrease with increasing flow velocity where at the same time mass transport from the water column toward biofilms increase (Beyenal and Lewandowski, 2002; Larned et al., 2004; Soininen, 2004). The algal composition of biofilms can adapt to the present flow conditions (Graba et al., 2013; Bondar-Kunze et al., 2016). With increasing flow velocities, the diversity of the diatom community may decrease because only specialized species can withstand (Soininen, 2004). It was reported that the EPS matrix gets thinner but denser (Wang et al., 2014), and that the production of EPS per bacterial cell increases with increasing flow velocity (Battin et al., 2003a). Besides flow velocity, near-bed turbulence is an important parameter shaping biofilm composition, architecture, and biomass (Labiod et al., 2007; Besemer et al., 2009; Risse-Buhl et al., 2017). While the flow velocity measures temporarily averaged flow, the turbulence kinetic energy (TKE) expresses the flow variations over time. These flow variations are even stronger close to solid boundaries like the stream bed and are thus considered an appropriate descriptor for the complex physical environment of streambed surfaces (Statzner et al., 1988).

Besides hydrodynamics, water quality (i.e., nutrients and chemical pollution) is an important environmental factor for community composition. In European rivers 960 organic chemicals were reported to be present by of which 42% are pesticides, 165 compounds are potentially hazardous to algae, and at least 7% inhibit photosynthesis (Busch et al., 2016). Targeting the Hill-reaction and decreasing the electron transport of the photosystem II (Shimabukuro and Swanson, 1969), these herbicides decrease primary production and result in changes in species richness and algal biomass after chronic exposure (DeLorenzo et al., 2001; Pesce et al., 2011).

During chronic exposure stressors exert a species selection pressure on the biofilm community. In the following sensitive species vanish and tolerant species dominate the community (toxicant-induced succession, TIS) resulting in an increase of the overall community tolerance. This principle is used in the SICT–approach (stress-induced community tolerance, Blanck et al., 1988; Tlili et al., 2015) and was evidenced for several toxicants (e.g., McClellan et al., 2008; Tlili et al., 2011).

The tolerance of stream biofilms toward herbicides differs due to the present species pool, which may have been pre-selected by previous stress exposures. This makes an interaction of combined stressors on community tolerance reliable. For instance, Rotter et al. (2013) and Schmitt-Jansen et al. (2016) showed that combined toxic and ionic stress result in a selection of unique communities impacting community tolerance. TIS was identified to be the main assembly rule behind induced community tolerance but other mechanisms like physiological acclimation are also conceivable (Schmitt-Jansen et al., 2016). Induced community tolerance may be especially of importance, when combined stressors result in co-tolerance patterns (Vinebrooke et al., 2004).

Besides the biofilm community, the biofilm matrix, respectively, the EPS, is known to interact with herbicides (Lawrence et al., 2001). The EPS matrix absorbs herbicides (Wolfaardt et al., 1994) and protects the biofilms against chemical stressors (Flemming and Wingender, 2010; Limoli et al., 2015).

As the above cited literature indicates that hydrodynamic conditions primarily control the biofilm composition in terms of species composition and EPS content (Ghosh and Gaur, 1998; Villeneuve et al., 2011b; Wang et al., 2014) we expect interactions between hydrodynamics and herbicide tolerance, as the EPS is also known to accumulate and protect the biofilm organisms from toxicans (Wolfaardt et al., 1994; Flemming and Wingender, 2010). The hypothesis that a higher biodiversity increases the community tolerance was tested under the “biological insurance hypothesis” (Yachi and Loreau, 1999) by Villeneuve et al. (2011b), already. While they did not link herbicide tolerance of biofilm communities to different flow velocities directly, they found a lower tolerance in a more heterogeneous and turbulent flow channel. Moreover, the relevance of near-bed turbulence and the EPS matrix as a potential mechanism of stressor interactions were not considered in their study.

We want to fill this gap of knowledge by addressing the questions whether (I) hydrodynamic-induced changes in species composition result in community tolerance to herbicides and how (II) the EPS content explains interacting effects of hydrodynamic and chemical stress in biofilms. In detail we hypothesize that:

- the herbicide tolerance of stream biofilms decreases with increasing near-bed flow velocity and turbulence
- the hydraulic stress increases the EPS biovolume of a biofilm, decreasing bioavailability of toxicants
- a hydrodynamic selection pressure changes community structure resulting in decreasing biodiversity and community tolerance.

## Materials and Methods

### Mesocosm Experiment

#### Mesocosm Setup

Biofilms were cultivated under spatially varying hydrodynamic conditions for 34 days in a hydraulic flume (length: 5.2 m, width 0.3 m). The flume was installed inside a “mobile aquatic mesocosm” (MOBICOS, Wollschläger et al., 2017) and constantly fed with water from the neighboring stream Selke. The catchment of the Selke is located in the Bode catchment (Harz, Germany) which is part of the Terrestrial Environmental Observations (TERENO), a dense, long-term monitoring program (Wollschläger et al., 2017). The stream reach next to the MOBICOS exhibits a mountainous character with coarse grained sediments and a near-natural flow regime.

**FIGURE 1:**
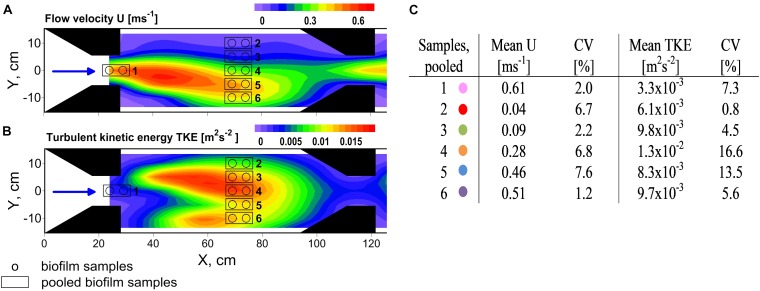
Top view on experimental flume section showing contour plots of the distribution of **(A)** flow velocity U and **(B)** turbulent kinetic energy TKE. The black arrows point out the flow direction. Several trapezoidal shaped installations (noozles) were used to contract the flow and create flow heterogeneity. Biofilms were sampled along a hydrodynamic gradient and pooled according to comparable hydrodynamic conditions. Mean values of U and TKE of pooled samples and their coefficient of variance (CV) are summarized in **(C)**. Colors indicate the samples for the residual figures.

Flow heterogeneity in the flume was created by several nozzles which contracted the flow and created varying zones of high and low flow velocity and TKE (Figure 1A). Biofilms were grown on large unglazed anthracite ceramic tiles. LED strips (SolarStringer SunStrip, daylight, Econlux, Cologne, Germany) supplied biofilms with a photosynthetic active radiation of 36.1 ± 5.8 μmol m^-2^ s^-1^ (mean ± SD) for 10 h/day during the 34 days of biofilm development.

The water level was controlled by a weir placed at the end of the flume and plateaued at about 16 cm at the sampling points. Discharge was monitored by a clamp-on ultrasonic flowmeter (MB 100H) installed at a tube section right before the flume entrance and was 8.5 L s^-1^. Abiotic parameters of the flume water, i.e., temperature, oxygen concentrations, pH, conductivity were measured by an EXO2 multiparameter probe (YSI Inc., Yellow Springs, OH, United States) at least once a week. At the same interval flume water was sampled for dissolved organic carbon (DOC), soluble reactive phosphorus (SRP), NO3-N, NH4-N, and Chl a (see Risse-Buhl et al., 2017 for methods description).

#### Biofilm Sampling

A custom made biofilm brush sampler adapted from Ritz et al. (2017) based on a toothbrush head within a sample chamber was used to sample the 34-day-old biofilms. Under a constant flow of sterile filtered stream water (0.2 μm) through the sample chamber realized by a peristaltic pump (Watson Marlow) at 2 mL s^-1^, the toothbrush head was placed on the biofilm patch, rotated and pushed three times. An area of 5.7 cm^2^ per patch was detached and transported with the flow of sterile water into the sampling tubes. Two neighboring patches per sample were pooled up to a total volume of 20 mL. Samples were stored at 8°C and further processed within the next 12 h. A total number of 12 samples was collected. Sample locations in the flume are shown in Figure 1. Additional biofilm samples for chlorophyll analysis were taken at different sampling spots to get a rough estimation of a possible relationship of chlorophyll a and hydrodynamic parameters (see Supplementary Figure 1).

#### Hydrodynamic Measurements

Three-dimensional current velocity measurements were conducted with a multi-static acoustic Doppler velocity ADV profiler (Vectrino II, Nortek AS, Norway) at 64 Hz for 5 min and 2.3 cm above the sampled biofilm patches. Velocity time-series were processed and the TKE was calculated based on the three dimensional variance of flow velocity according to Risse-Buhl et al. (2017). Flow velocity was calculated as $U=\frac{1}{N}\sum_{i=1}^{N}u_{i}$ where *u* and *N* denote the longitudinal component of the velocity vector and the number of measurements at each location, respectively. Overall, 32 measurements were conducted above the biofilm samples and in their vicinity. Resampling of point measurements on a regular grid was done using a multiquadratic radial basis function with an anisotropic ratio of 3 (Golden Software Surver v9.2.397).

#### Assessing Stress-Induced Community Tolerance (SICT)

The herbicide Prometryn was selected for chemical exposure because environmentally relevant concentrations in the lower nmol range were recently found in a near-by catchment (Rotter et al., 2015). In this study community tolerance induced by Prometryn was evidenced during *in situ* investigations using biofilms grown directly in the river (Rotter et al., 2015).

Using 96-well plates, aliquots of 150 μL of the biofilm samples were taken under constant stirring to ensure homogeneity. Biofilm suspensions were incubated with Dimethylsulfoxid (DMSO)-dissolved Prometryn at six concentrations within a 10-fold dilution series ranging from 0.00084 to 83.8 μmol L^-1^. Each dilution step of the herbicide and a solvent control (final DMSO concentration: 0.05%, not showing effects in a former study; Rotter et al., 2011) were used in triplicate. The 96-well plates with the biofilm-herbicide suspension were placed under constant light (130 – 150 μmol photons m^-2^ s^-1^) and intense shaking at 350 rpm (KS250basic, IKA Labortechnik, Staufen, Germany). Inhibition of variable chlorophyll fluorescence was analyzed using an Imaging Maxi pulse-amplitude modulated (PAM) fluorimeter (Walz, Effeltrich, Germany; instrumental settings: Intensity 4, Gain 4, Damping 2). After 1 h of incubation biofilms were dark adapted for 5 min and the variable chlorophyll fluorescence was measured three times. After applying a saturation pulse the maximum chlorophyll fluorescence at dark adapted conditions (Yield I) was assessed following Schmitt-Jansen and Altenburger (2008).

#### Extraction and Analysis of EPS Fractions

Extracellular polymeric substances were extracted according to Barranguet et al. (2004). Briefly, 2 mL biofilm samples were resuspended in bi-distilled water, vortexed and centrifuged (5 min, 3500 rpm). The supernatant was collected and represents the soluble EPS fraction. The bound EPS fraction was extracted from the residual pellet after incubation at 95°C for 30 min in 2 mL 0.1 M H_2_SO_4_. Both fractions were analyzed for four groups of substances by comparing the spectrophotometric measurements to the ones of the standard substances. The carbohydrate content of the EPS was quantified according to the phenol-sulphuric acid assay by DuBois et al. (1956) with D-Glucose (Sigma-Aldrich, CAS 50-99-7) as a standard. For protein and humic acid analysis the Folin phenol assay by Lowry et al. (1951) was used. Results had to be corrected due to interferences of humic acids after Frolund et al. (1995). Bovine Serum Albumin (Biorad, CAS n.a.) was used as standard substance for the protein assay, and humic acid (Roth, CAS 1415-93-6) for the humic acid assay. A hydroxydiphenyl assay by Blumenkrantz and Asboe-Hansen (1973) and its modification by Kintner and Van Buren (1982) was used to assess the uronic acid content. Glucoronic acid (Sigma, CAS 6556-12-3) was used as the standard substance.

#### Preparation and Taxonomic Assessment of Diatoms

To identify diatoms, which were the dominating algae fraction within the biofilms [71% according to measurements derived from a Phyto-PAM fluorimeter (Walz, Effeltrich, Germany)], organic material was removed by boiling the biofilm suspension first in hydrochloric acid and second in hydrogen peroxide solution (30%) until the solution was colorless. Samples were washed in water during 24 h after each boiling step. Species were identified using a light microscope (×630 zoom, Leica DMI4000B with a PFC250 camera, Leica Microsystems GmbH, Wetzlar, Germany) based on their microscopic siliceous exoskeleton. At least 400 valves per sample were counted. Hofmann et al. (2013) was used for the taxonomic determination of the diatoms to species level as far as possible.

#### Data Analysis and Assessment

Concentration-response curves were modeled and effective concentrations that induced 50% of inhibition of the photosynthetic yield (YI; EC50) were calculated using the software SigmaPlot 13.0 (Systat Software GmbH, Erkrath, Germany). Concentration-response curves were modeled based on the four-parametric Hill-equation y = y_o_ + (ax^b^)/(c^b^ + x^b^) (y_o_ = the minimum value of photosynthesis inhibition fixed at 0.0001%, *a* = the maximum value of photosynthesis inhibition fixed at 100%, *c* = the inflection point (here the EC50), *b* = Hill’s slope at point *c*) with 1000 iterations per curve. Correlations of the EC50-values with the other parameters were tested via Spearman-test.

The diatom community diversity and evenness was assessed with the Shannon-Index (H_s_) and the Pielou-Index (E_H_), respectively:

$$H_{S}=-\sum_{i=1}^{S}p_{i}\times \log\;p_{i}\;\;\;\;\;\mathrm{with}\;\;\;\;\;\;p_{i}=\frac{n_{i}}{N}\;\;\;\;\;\;E_{H}=\frac{H_{S}}{H_{\max}}$$

*S* = number of present species, *N* = total number of individuals (400), *n_i_* = number of individuals per species, *p_i_* = relative abundance of the i-species, *H*_max_ = maximal possible Shannon Index.

A correspondence analysis (CCA) was performed based on the hydrodynamic data and the taxonomical dataset of the diatom community. To obtain a more robust dataset the species of the genera *Navicula, Nitzschia* and *Fragilaria* were pooled according to their genera. The CCA was performed using the software R version 3.5.0 and the *vegan* R package (Oksanen et al., 2015; R Core Team, 2018).

### Herbicide Toxicity Modulation by Artificial EPS

Based on the results of the mesocosm experiment using natural communities a second experiment was performed using a benthic diatom culture to confirm the role of EPS on the response of biofilms to Prometryn. Therefore, *Nitzschia palea* biofilms (SAG Göttingen, Strainnumber 1052-3A) were grown in 24-well plates (initial cell count 1 × 10^6^ cells mL^-1^). After 3 days of growth, the diatom culture medium was exchanged with five different artificial EPS-enriched diatom culture media. Briefly, the diatom medium was enriched by one of the four individual EPS fractions studied previously (humic acid, carbohydrates, proteins, uronic acid, see section “Extraction and Analysis of EPS Fractions”). For each of the four media, four different enrichment concentrations were tested such as 0, 25, 50, 100 mg L^-1^ (for details on the calibration curve see Supplementary Table 2). In parallel, a medium containing a mixture of all four EPS fractions (composed according to the EPS fractions found in the biofilms of the mesocosms experiment: carbohydrates 28.9%, proteins 30.9%, humic acids, 25.9%, uronic acids 4.3%) was tested at five different enrichment concentrations: 0, 50, 100, 200, and 400 mg EPS L^-1^. For each of these media (four single fractions of EPS^∗^four EPS concentrations and one mixture of fractions^∗^five EPS concentrations), the inhibition of photosynthesis of *N. palea* biofilms was assessed after exposure to six Prometryn concentrations ranging from 0.00026 to 83.2 μmol L^-1^ (solved in 0.1% DMSO). The inhibition of chlorophyll fluorescence after dark adaptation (Yield I) was measured after 1 h of exposure as described above.

## Results

### The Mesocosm Experiment

#### Hydrodynamic Conditions

Flow velocities and TKE varied over one order of magnitude and ranged from 0.04 to 0.62 m s^-1^ and from 3.2 × 10^-3^ to 1.4 × 10^-2^ m^2^ s^-2^, respectively, covering the full range of near-bed flow velocities and the upper range of TKE that was found in the neighboring stream Selke (Risse-Buhl et al., 2017). Highest flow velocities were observed right at the nozzles exit whereas highest TKE values coincided with the centerline (*Y* = 0 cm, Figure 1B) of the flume, in-between the two sets of nozzles. Whereas flow velocities and turbulence in natural streams are positively correlated because both are predominately linked to riverbed roughness, in the hydraulic flume the causes of high and low flow velocities and origin of turbulence are associated to the contraction of the flow and shear layers due jet-like flow. However, properties of flow velocity and TKE result in comparable forces at small spatial scales and therefore show similar effects on biofilms. The coefficients of variance (CV) of flow velocities and TKE values of pooled biofilm samples were small and, except for the two highest TKE values, below 15% (Figure 1C). In the following it will be exclusively referred to the mean values of flow velocities and TKE. For results for physico-chemical parameters see Supplementary Table 1. For additional information on Chl a distribution see Supplementary Figure 4.

#### Biofilm Tolerance Toward Prometryn

**FIGURE 2:**
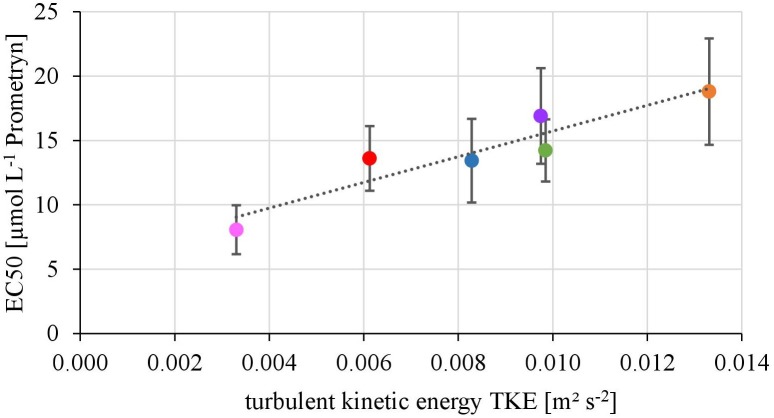
Relationship of the EC50-values derived from toxicity testing of biofilms, quantified as inhibition of the maximum photosynthetic Yield after 1 h of exposure and turbulent kinetic energy TKE (*R*^2^ = 0.87, *p* < 0.05). The error bars of the EC50-values represent the calculated standard error after modeling of concentrations-response curves using the Hill model.

The EC50-values of photosynthesis inhibition of biofilms toward Prometryn ranged from 8.06 ± 1.90 to 18.80 ± 14.23 μmol L^-1^. The yield and TKE showed a significant linear correlation with *R*^2^= 0.88 (*p* < 0.05) (Figure 2). The lowest EC50 values were detected at lowest TKE and the highest EC50 values for the highest TKE. However, no linear correlation with the flow velocity was found (*R*^2^= 0.17, *p* = 0.33, Supplementary Figure 2).

#### EPS Matrix of Natural Communities at Contrasting Hydrodynamic Conditions

**FIGURE 3:**
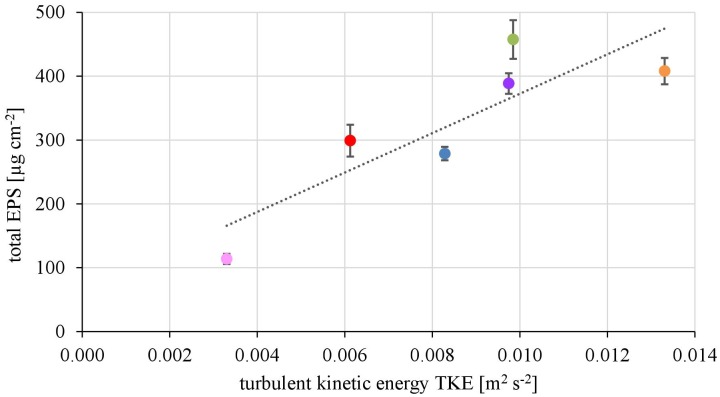
The total EPS biovolume (μg cm^2^) shows positive correlation with increasing turbulent kinetic energy TKE (*R*^2^ = 0.74, *p* < 0.05, *n* = 3).

The EPS of the biofilms from contrasting hydrodynamic conditions mostly consisted of carbohydrates, proteins and humic acids at an almost equal mean percentage share of 37.5 ± 2.9, 29.2 ± 2.0, and 28.6 ± 3.3%, respectively. Uronic acids made up only a marginal fraction of the EPS (4.75 ± 0.39%). The total amount of EPS ranged from 113 ± 8 μg cm^-2^ at the lowest TKE value to 458 ± 30 μg cm^-2^ at the highest TKE value. A positive linear correlation (*R*^2^= 0.76) of the total EPS biovolume and TKE was found and confirmed by a Spearman-test (Figure 3, *p* < 0.05). Furthermore, the total EPS correlated positively with the EC50-values of biofilms toward Prometryn (*R*^2^ = 0.74, *p* < 0.05). No correlation with the flow velocity was found (*p* = 0.16, Supplementary Figure 3).

#### Diatom Community Composition and Biodiversity

**FIGURE 4:**
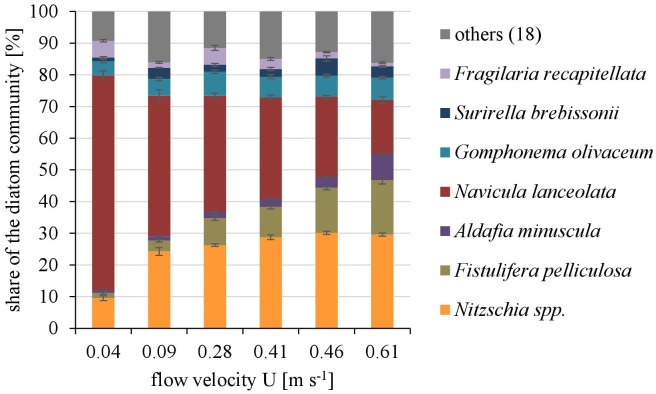
The dominating taxa of the diatom community along a gradient of flow velocities U. Mean ± SD (*n* = 3) values of each taxa with a percentage share ≥5% of the total diatom community are shown, the other eighteen taxa are pooled as “others.”

**FIGURE 5:**
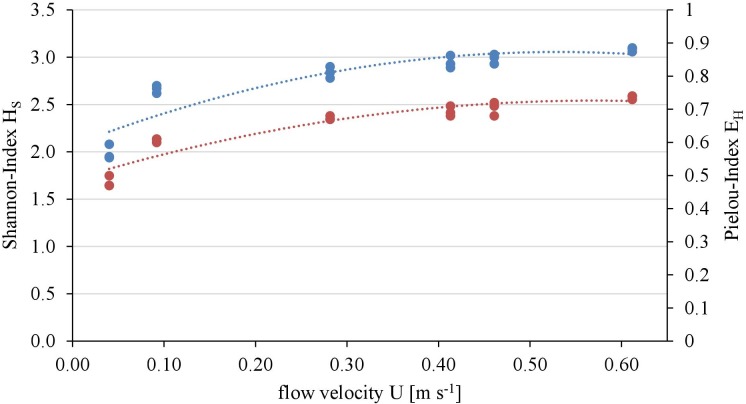
Relationship of taxonomic diversity of the diatom communities expressed as Shannon-Index (blue, polynomial relationship: *R*^2^ = 0.82) and evenness expressed as Pielou-Index (red, polynomial relationship: *R*^2^ = 0.88) with the flow velocity.

**FIGURE 6:**
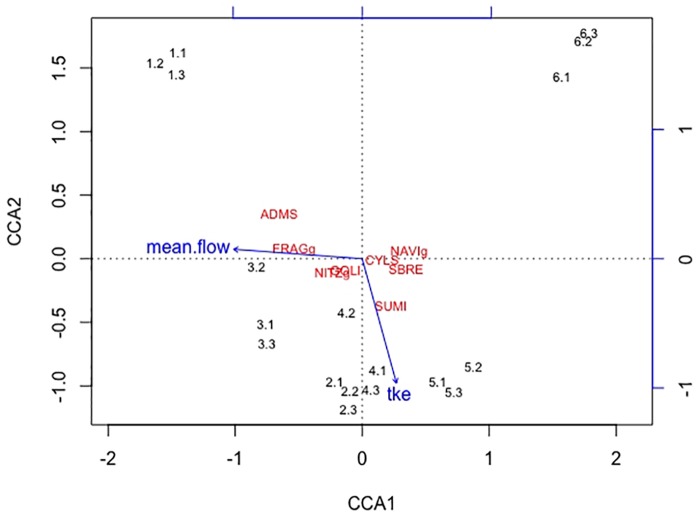
The first axis CCA1 explains 91% of the differences found in the diatom community and the second axis CCA2 describes 8.9% of the differences. Blue arrows indicate the direction of the two increasing hydrodynamic parameters (mean flow, TKE) within this cluster. Numbers represent the diatom samples and the respective replicates. The diatom species *Adlafia minuscula* (ADMS), *Fragilaria spp*. (FRAGg), *Nitzschia spp*. (NITZg), *Gomphonema oliveacum* (GOLI), *Cyclotella spp*. (CYLS), *Navicula spp*. (NAVIg), *Suriella brebissonii* (SBRE), and *Suriella minuta* (SUMI) are shown within the CCA cluster. The diatom community aligns with the flow velocity, only one species also partly aligns with TKE.

Overall twenty-five diatom taxa were found, from which seven taxa made up more than 5% of the total diatom community, respectively. The diatom community changed along the gradient of the flow velocity (Figure 4). The taxonomic diversity and evenness increased with increasing flow velocity (Figure 5). At lower flow velocities *Navicula lanceolata* was dominating with an approximate percentage share of 70%. At higher velocities the percentage share of *N. lanceolata* decreased to 20% at 0.61 m s^-1^ and favored other species as *Nitzschia spp., Fistulifera pelliculosa* and *Adlafia minuscula*. Diatom taxa were more equally distributed at higher flow velocities. No clear trends in dependence on TKE were found. Shannon-Index and Pielou-Index show a correlation with the flow velocity (*p* < 0.05) but not with the TKE. The CCA showed a high explanatory value for the first axis (91%) and a lower one for the second axis (8.9%) (Figure 6). Samples were ordered along the first axis from the right to left side in accordance with the increase of the flow velocity but were also displayed from the top to the down of the plot in accordance to TKE values. The ordination plot displays the importance of both flow parameters spanning a two-dimensional space in which the diatom community developed. However, axis eigenvalues highlighted the importance of flow velocity over TKE in community structure.

### Herbicide Inhibition Through Artificial EPS

**FIGURE 7:**
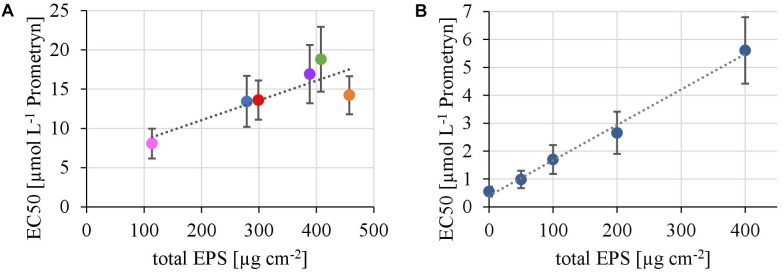
**(A)** Correlation of the EC50 values derived from toxicity testing of flume biofilms, quantified as inhibition of the Yield I after 1 h of incubation, with their total biovolume of the EPS matrix (*y* = 0,0251*x* + 6,04, *R*^2^ = 0.71, *p* < 0.05). **(B)** Correlation of the EC50 values derived from toxicity testing of biofilms of *N. palea*, quantified as inhibition of the Yield I after 1 h of incubation, with the total mass of additional EPS (*y* = 0.0127*x* +0.4, *R*^2^ = 0.99, *p* < 0.01).

**FIGURE 8:**
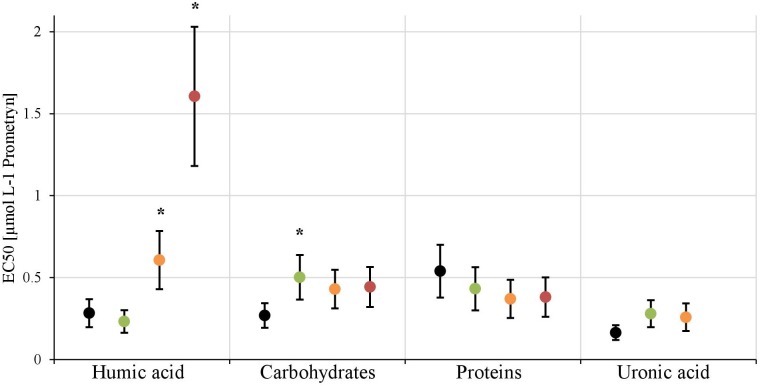
The EC50-values derived from the inhibition of photosynthethic Yield I after 1 h are shown for each treatment [control (black), +25 mg L^-1^(green), +50 mg L^-1^(orange), +100 mg L^-1^(red)] of the four EPS fractions. The EC50-value for the 100 mg L^-1^ treatment of uronic acid couldn’t be calculated. Significant differences toward the control, respectively, the next lower concentration, are marked with a^∗^.

In the first experiment we observed a positive correlation of the total EPS with the EC50-values (Figure 7A), in the second experiment, a highly significant positive correlation between the mean EC50-values of *N. palea* biofilms toward Prometryn and the total artificial EPS-content was found with *R*^2^= 0.99 (*p* < 0.01) (Figure 7B). For the individual EPS fractions, only humic acids had a significant positive correlation with the EC50-values (*R*^2^= 0.90) (Figure 8). Other fractions showed comparable trends in the EC50-values, but no significant relationships were found.

## Discussion

### Herbicide Tolerance of the Biofilm

The herbicide tolerances positively correlate with near-bed turbulence but not with flow velocity. Villeneuve et al. (2011a) found that an increasing flow velocity has a positive effect on periphyton primary production, algal density, bacterial production, and bacterial density. This may be caused by an increased retention of nutrients by the biofilm at high flow velocities (Biggs and Hickey, 1994; Villeneuve et al., 2011a). In a follow-up study, Villeneuve et al. (2011b) tried to link these structural and functional periphyton responses to changes in tolerance toward a PSII-inhibiting herbicide but could not find a correlation of the EC50-values and flow velocity (A. Bouchez, personal communication). This finding was partly confirmed by our study; however, the clear correlation with TKE indicates the relevance of the temporal velocity variations for changes in the biofilms. In conclusion, we could not confirm our first hypothesis that increasing flow velocity and turbulence decreased the herbicide tolerance. Instead, we found that in particular turbulent conditions induce a higher tolerance toward herbicides.

In a recent study, Risse-Buhl et al. (2017) highlighted the role of the near streambed turbulence on the composition and architecture of stream biofilms: The algal biovolume as well as the surface coverage of biofilms matured in streams increased with increasing near streambed turbulence. This leads us to the conclusion that TKE-induced changes in the composition and architecture of the biofilms is the main factor for stress-induced community tolerance toward the herbicide. Therefore, the EPS matrix as well as the diatom community composition were analyzed in more detail in this study to gain insights in potential mechanisms of stressor interactions.

### Changes of the EPS Matrix

Similar to the EC50-values of biofilms toward herbicides, the changes in total EPS content of the biofilm matrix increased with near-bed turbulence but not with flow velocity thereby partly confirming our second hypothesis. However, evidence from literature on this relationship remains unclear. The total EPS content of bacterial biofilms grown in flow cells either increased with laminar flow (Percival et al., 1999; Araújo et al., 2016), or with turbulence (Simões et al., 2007), whereas Wang et al. (2014) found a positive correlation of the bound EPS with flow velocity. Increasing flow velocity and turbulence were not found to be responsible for changes of the total EPS content of autotrophic biofilms grown in flumes (Battin et al., 2003a) but for changes of the EPS production per cell. The opposite was found in the Selke stream, where less glycoconjugates were produced per microbial cells but overall the biovolume of glycoconjugates increased with TKE (Risse-Buhl et al., 2017). Carbohydrates or polysaccharides of the EPS matrix of bacterial biofilms are shown to be important for building a mature 3-dimensional biofilm structure (Limoli et al., 2015). In combination with proteins that have the affinity to crosslink to, e.g., carbohydrates, complexes are formed that substantially contribute to the stability of the biofilm matrix (Fong and Yildiz, 2015). The increasing carbohydrate and protein content in our flume biofilms indicates an increased stability of the biofilms 3-dimensional structure at increased TKE. Thus, the EPS seems to help buffering hydrodynamic forces affecting the biofilm community (Biggs and Hickey, 1994; Freeman and Lock, 1995). EPS, as such, is still a black box since it is composed of a large number of diverse substances, ranging from carbohydrates, glycoconjugates, peptides, humid acids, uronic acids to nuclides (Flemming et al., 2016). The functions of these substances are far from being completely understood. Up to now, there are no consistent trends on the EPS content of biofilms at different hydrodynamic conditions in literature. However, none of these studies analyzed TKE as a driving factor of the EPS content. Our study clearly shows that TKE is the prominent factor for changes in the biofilm EPS content.

### Influence of the EPS Matrix on Toxicity

The results of the first, and especially the second, experiment illustrate a lower sensitivity of biofilms toward the herbicide at high humic acid concentrations. We hypothesize form these findings that the EPS matrix binds the toxicants changing their bioavailability to the organisms. Indeed, binding mechanisms for herbicides have been described for DOC in soils, for long (Baskaran and Kennedy, 1999; Ling et al., 2006). Furthermore, the absorption of herbicides to humic acids, which is one fraction of the DOC pool in soils and aquatic systems, is well described in literature (Bollag and Myers, 1992; Senesi, 1992). Out of all binding mechanisms, e.g., hydrophobic adsorption or covalent binding, adsorption is considered to be the most important interaction (Senesi, 1992). For aquatic ecosystems, the accumulation (Lawrence et al., 2001) and the sorption (Headley et al., 1998) of organic micropollutants (including herbicides) in the EPS matrix was already shown, but not linked to changes in bioavailability and toxic effects. In our study, we observed that the EPS matrix of biofilms lowers the negative effect of Prometryn (increase of EC50-values based on photosynthesis inhibition) which could be regarded as an indirect evidence of changes in bioavailability. This result represents a first step toward a better understanding of biofilm matrix effects and the role of toxicokinetic processes for algae toxicity. To prove this mechanism in more detail, further experiments with chemical analysis at multiple time points are needed.

### Diatom Community Structure and Diversity

According to our third hypothesis we found a clear trend in both community structure and taxonomic diversity parameters (Shannon Index & Pielou Index) in dependence to flow velocity while TKE seems to have no effect. The increase in biodiversity with increasing flow velocity is contradictory to other studies. Soininen (2004) found a decrease in the diatom diversity (Shannon Index) over a gradient of three flow velocities (0.1, 0.4, and 1.0 m s^-1^). Villeneuve et al. (2011b) conclude that the heterogeneity of hydraulic conditions increases the overall biodiversity by creating more microhabitats for a diverse community, but cannot correlate the biodiversity with a gradient of these conditions.

Multiple reasons may be responsible for the distribution of the diatom community, e.g., increased hydrodynamic shear forces leading to an adaption of the community. Certain species would be better adapted to these conditions and thrive while others vanish.

Whether the individual sensitivity of single species has an influence on the observed changes in biofilm tolerance cannot be concluded, as there are not enough species-specific autoecological data available. Considering diversity on its own, the “biological insurance hypothesis” (Yachi and Loreau, 1999) applied on our diversity results suggest that the biofilms at higher flow velocities would be less sensitive toward herbicides. Instead we confirm results by Villeneuve et al. (2011b), who also could not confirm Yachi’s hypothesis on the herbicide application on biofilms in the first place and indicate that other mechanisms than diversity are responsible for the tolerance patterns, found in these studies.

Furthermore, we found no evidence that the biofilm diversity has an influence on the herbicide tolerance of the biofilm and could not prove our third hypothesis that the diatom diversity decreases due to the hydrodynamic selection pressure. Nevertheless, only assumptions can be made based on our results and the complex interaction thought to be present. We suggest that more complex processes shape the diversity than originally thought. In particular, the varying effects of flow velocity and turbulence on biofilm diversity and underlying mechanisms need to be the focus of future studies.

## Conclusion

Our results clearly showed interacting effects of hydrodynamics and toxic stress which are frequently co-occurring in European waters. Nõges et al. (2016) revealed a frequency of 10–25% of co-occurrence of hydrological (with potential effects on hydrodynamic conditions) and toxic stressors in reported data sets from rivers and transitional and coastal waters, however, these numbers may be even higher, as toxicants are not frequently included in these analyses (Schäfer et al., 2016). Additionally, interacting effects from hydrological and toxic stress may be especially important for streams with steep gradients in altitude or high morphological degradation.

The stress-induced tolerance of the biofilm communities toward a herbicide, found in this study and the causal findings behind stressor interactions point to an antagonistic effect at community level. Côté et al. (2016) revealed that antagonistic effects are especially being observed at community level whereas other levels of biological complexity (e.g., population of sub-cellular studies) mainly showed additive or synergistic interactions. While laboratory studies mainly exclude the invasion of other species with higher tolerances to the selecting stressors, these experimental settings may bias the findings toward additive of synergistic effects. This is further demonstrating the importance of *in situ* studies mimicking the natural situation as close as possible. The experimental system used in this study allowed manipulation of the stressor gradients but also species succession processes from the bypass stream, which is an important response mechanism of natural communities toward combined stress.

Our approach studied two dominant mechanisms behind the interacting effects: while changes in the community structure is frequently found to be the cause for SICT, in this study the role of EPS and its potential relevance for the bioavailability of toxicants was investigated. This hints to the complexity of stressor interactions which could on the one hand act on the assembly rules of communities resulting in changes on species sensitivity, diversity and functioning but also on the susceptibility of communities to a stressor, in this case changes in the toxicokinetic processes. This mode of interaction may not only be important for chemical pollution but also for other factors like dissolved organic material or nutrients. Transferring these small-scale interactions to a larger scale many hydro-morphologically altered streams and rivers in Europe pose a risk for lower tolerances of the phototrophic benthic community in streams.

## Data Availability Statement

The raw data supporting the conclusions of this manuscript will be made available by the authors, without undue reservation, to any qualified researcher.

## Author Contributions

BP did the lab work and statistical analysis and wrote the draft. CA and UR-B contributed to the conception and design of the mesocosm, maintained the mesocosm during the experiments and wrote sections of the draft. CA conducted and analyzed the hydrodynamic measurements and provided the figures. UR-B took the biofilm samples within the mesocosm and provided data for physico-chemistry of flume water and Chl a of biofilms. TH, MW, and MS-J provided substantial feedback and revision of the draft. FL contributed to the analysis of the diatom community (CCA) and their interpretation. MW contributed to the conception and design of the mesocosm and had strong impact on the abstract. MS-J and TH supervised the project. MS-J initiated this study and designed the tolerance and confirmation experiment including the structural and functional descriptors of the biofilm community. All authors contributed to the submitted draft with overall feedback, insightful discussions, and improvements on the text.

## Conflict of Interest Statement

The authors declare that the research was conducted in the absence of any commercial or financial relationships that could be construed as a potential conflict of interest. The handling Editor declared a past co-authorship with several of the authors FL and MS-J.
