# Supplementary material for: Hydrodynamics Alter the Tolerance of Autotrophic Biofilm Communities Toward Herbicides
Source: Front Microbiol. 2018 Dec 4;9:2884. doi: 10.3389/fmicb.2018.02884 (PMC6288176; doi:10.3389/fmicb.2018.02884)
Supplement: Supplementary file 1 [file Data_Sheet_1.docx]

Supplementary Material

Hydrodynamics alter the tolerance of autotrophic biofilm communities toward herbicides

Bastian H. Polst^1,2,3*^, Christine Anlanger^4,5^, Ute Risse-Buhl^4^, Floriane Larras^1^, Thomas Hein^2,3^, Markus Weitere^4^ and Mechthild Schmitt-Jansen^1*^

^1^ Department of Bioanalytical Ecotoxicology, Helmholtz-Centre for Environmental Research - UFZ, Leipzig, Germany ^2^ Institute of Hydrobiology and Aquatic Ecosystem Management, University of Natural Resources and Life Sciences, Vienna, Austria ^3^ WasserCluster Lunz, University of Natural Resources and Life Sciences, Lunz, Austria ^4^ Department of River Ecology, Helmholtz-Centre for Environmental Research - UFZ, Magdeburg, Germany, ^5^ Institute for Environmental Sciences, University of Koblenz-Landau, Landau, Germany

**Correspondence:**^x^Corresponding Authors
bastian-herbert.polst@ufz.de

Mechthild.Schmitt@ufz.de

# Supplementary Table

Table 1: The range of the results of the measurements of the physico-chemical Parameters within the mesocosm are shown. Overall sample size is shown as “n”.

| Paramter | Unit | n | Range (min - max) |
| --- | --- | --- | --- |
| Temperature | °C | 5 | 5.8 - 11.2 |
| Oxygen concentration | mg l^-1^ | 5 | 11.4 - 13.8 |
| Oxygen saturation | % | 5 | 104 - 118 |
| pH |  | 5 | 7.7 - 8.2 |
| Conductivity | µS cm^-1^ | 5 | 344 - 393 |
| DOC | mg l^-1^ | 6 | 1.8 - 3.6 |
| NO_3_-N | mg l^-1^ | 6 | 0.8 - 2.1 |
| NH_4_-N | mg l^-1^ | 7 | < 0.01 - 0.04 |
| SRP | mg l^-1^ | 6 | 0.01 - 0.02 |
| Chl a | µg l^-1^ | 5 | 2.6 - 7.4 |

**Table 2**: Range [mg L^-1^ & µg cm^-2^] of the calibration curves used in 2.1.5 for the determination of the EPS content. R² is given for the linear correlation of the measured absorption values of the calibration curve.

|  | Carbohydrates | | Proteins | | Humic acids | | Uronic acids | |
| --- | --- | --- | --- | --- | --- | --- | --- | --- |
|  | mg L^-1^ | µg cm^-2^ | mg L^-1^ | µg cm^-2^ | mg L^-1^ | µg cm^-2^ | mg L^-1^ | µg cm^-2^ |
|  | 0 | 0 | 0 | 0 | 0 | 0 | 0 | 0 |
|  | 25 | 43.86 | 20 | 35.09 | 10 | 17.54 | 5 | 8.77 |
|  | 50 | 87.72 | 40 | 70.18 | 20 | 35.09 | 10 | 17.54 |
|  | 100 | 175.44 | 80 | 140.35 | 40 | 70.18 | 20 | 35.09 |
|  | 150 | 263.16 | 120 | 210.53 | 60 | 105.26 | 30 | 52.63 |
|  | 200 | 350.88 | 160 | 280.70 | 80 | 140.35 | 50 | 87.72 |
|  |  |  | 200 | 350.88 | 100 | 175.44 | 80 | 140.35 |
|  |  |  |  |  | 200 | 350.88 |  |  |
| R² | >0.99 | | >0.99 | | >0.99 | | >0.99 | |

# Supplementary Figures

2.1 Supplementary chlorophyll data: Sampling

Samples for a Chl a analysis with high-performance liquid chromatography (HPLC) were taken at different sample spots as the other samples, which were used for the herbicide assay, the EPS and the community analysis. The position of the samples within the flume is shown in figure 1. Chl a of biofilm samples was trapped on glass fiber filters, extracted with ethanol and several freezing/thawing cycles and measured by high performance liquid chromatography (Dionex, Thermo Fisher Scientific Corporation, Waltham, MA, USA).


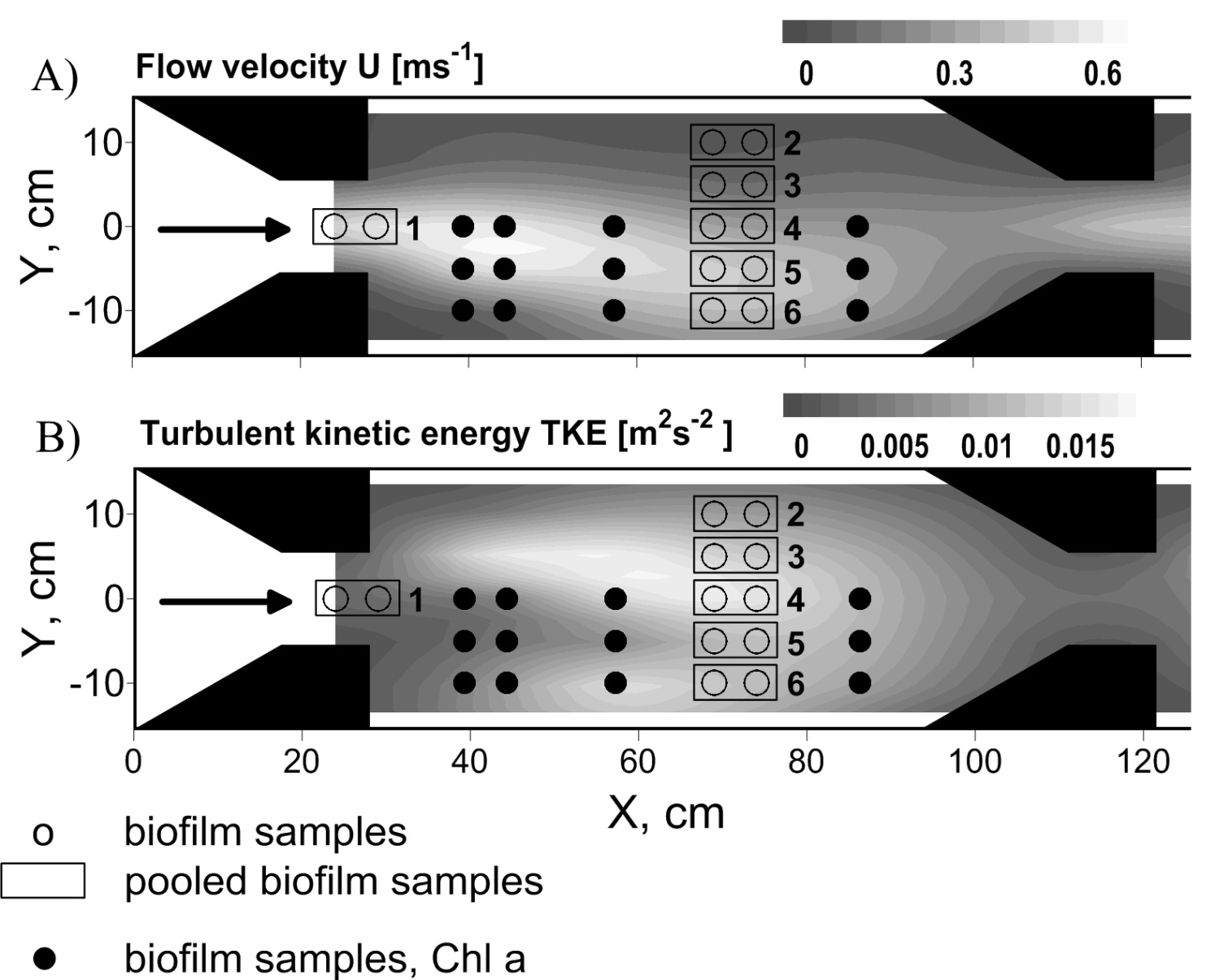


**Figure 1**: Top view on experimental flume section showing contour plots of the distribution of (a) flow velocity U and (b) turbulent kinetic energy TKE. The black arrows point out the flow direction. Several trapezoidal shaped installations (noozles) were used to contract the flow and create flow heterogeneity. Biofilm samples for the herbicide assay are shown as empty circles and their sample number is indicated by numbers 1 to 6. Biofilm samples for the Chlorophyll a analysis with the HPLC are shown as black dots.

2.2 Additional figures showing relationships of EC50-values and EPS with flow velocity

For the sake of completeness, SI figure 2 and SI figure 3 of the supplemental information show the relationship of the EC50-values and the total EPS with the flow velocity as a counterpart for figure 2 and figure 3 of the original manuscript.

**
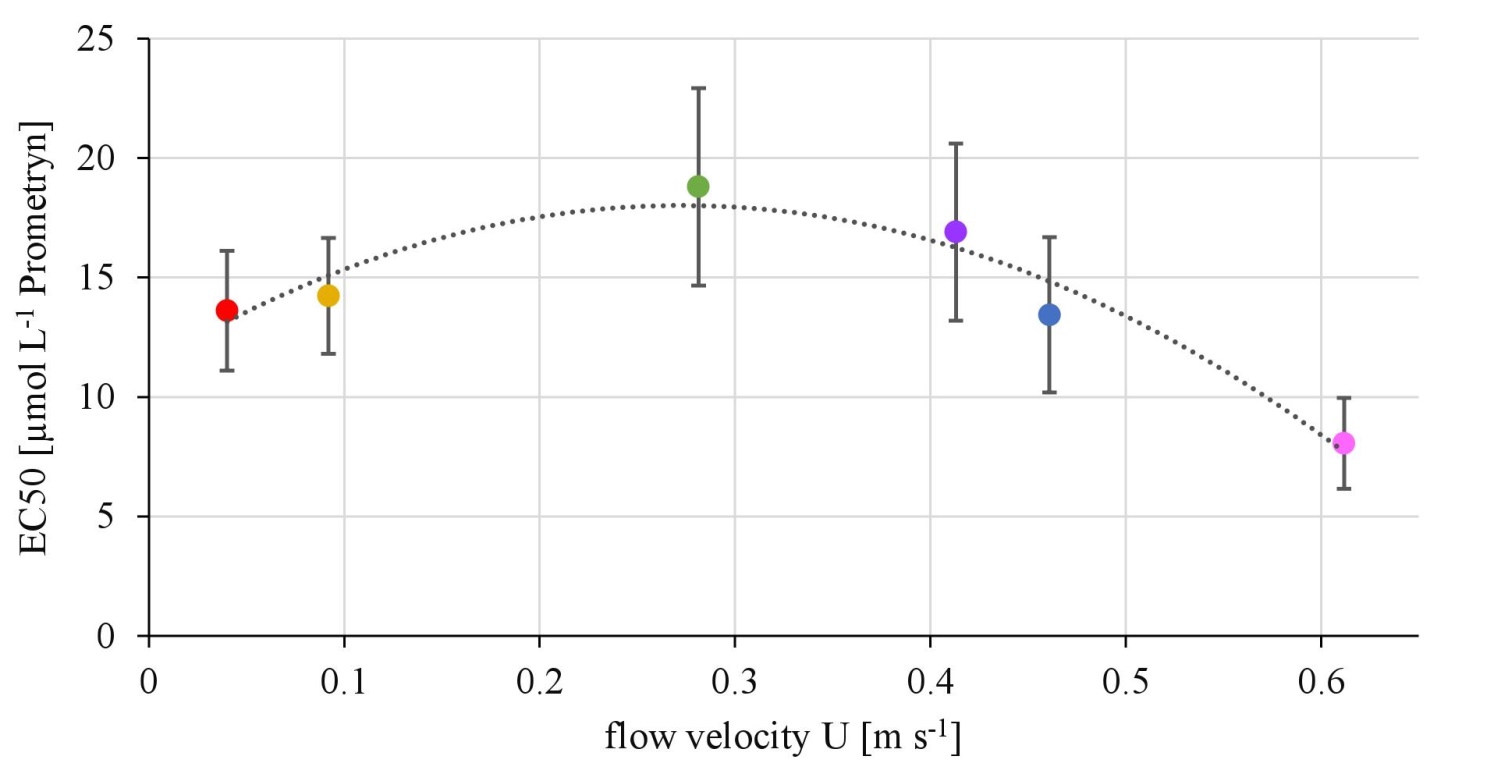
**

**Figure 2**: Relationships of the EC50-values derived from toxicity testing of biofilms, quantified as inhibition of the maximum photosynthetic Yield after one hour of exposure and mean flow velocity. The error bars of the EC50-values represent the calculated standard error after modelling of concentrations-response curves using the Hill model.


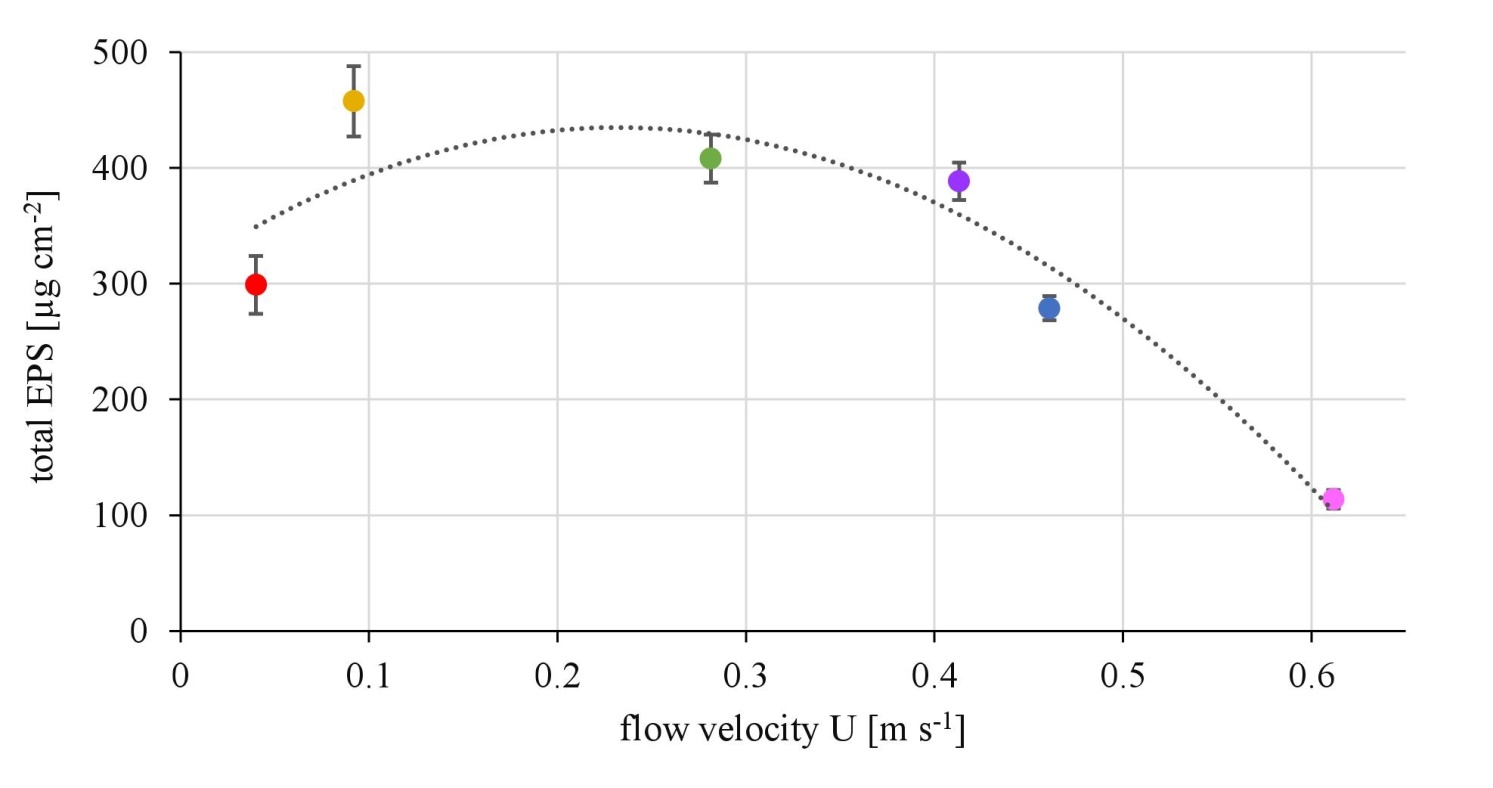


**Figure 3**: Relationship of the total EPS biovolume, quantified as the sum of the four unique fractions, and the mean flow velocity. The error bars represent the standard deviation of the triplicates.

2.3 Supplementary chlorophyll data: Results

A significant negative correlation of the Chl a concentrations and the mean flow velocity U was found (p=0.008) (Fig. 4a). However, no correlation of the Chl a concentrations was found with TKE (p>0.05, Fig 4b).


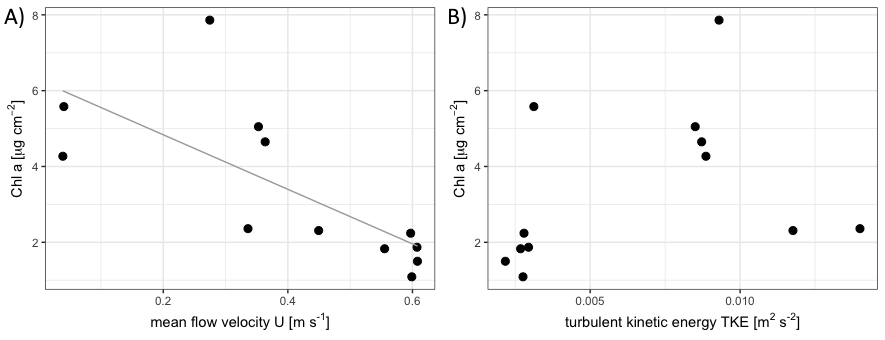


**Figure 4**: Relationship of the chlorophyll a concentration with the flow velocity U (A) and the turbulent kinetic energy is shown (B) (N=12).

The correlation of the Chl a with the mean flow velocity U confronted the correlation of the EPS with the TKE. Furthermore, it supported the correlation of the diatom diversity with the mean flow velocity. Therefore, we can conclude that higher mean flow velocities came along with a change in the biofilm community composition, decreasing diatom diversity and their chlorophyll content, respectively the density of algae cells overall. Still, this does not seem to have an effect on the EPS content, as the EPS shows no correlation with the clow velocity at all but increased with TKE. Thus, an increased EPS production per algal biomass seems likely as already reported by Battin et al. (2003a).

Battin, T.J., Kaplan, L.A., Newbold, J.D., Cheng, X.H., and Hansen, C. (2003a). Effects of current velocity on the nascent architecture of stream microbial biofilms. *Applied and Environmental Microbiology* 69(9)**,** 5443-5452. doi: 10.1128/aem.69.9.5443-5452.2003
